# Evaluation of the modified colorimetric resazurin microtiter plate-based antibacterial assay for rapid and reliable tuberculosis drug susceptibility testing

**DOI:** 10.1186/s12866-014-0259-6

**Published:** 2014-10-07

**Authors:** Victoria Katawera, Mark Siedner, Yap Boum II

**Affiliations:** Epicentre Mbarara Research Centre, Mbarara, Uganda; Mbarara University of Science and Technology, Mbarara, Uganda; Massachussetts General Hospital and Harvard Medical School, Boston, USA

## Abstract

**Background:**

The resazurin microtiter assay (classic REMA), a colorimetric liquid culture-based drug susceptibility assay for *Mycobacterium tuberculosis* (MTB), has been endorsed by the World Health Organization. The assay requires 8-16 days to obtain results, delaying management of drug resistant tuberculosis patients. A modified REMA which allows results in as little as 24 hours for bacterial strains, has been developed and validated using *Staphylococcus aureus,* but has not yet been evaluated for MTB*.* Therefore we assessed the performance of the modified REMA for rifampicin (RIF) and isoniazid (INH) susceptibility, using the classic REMA as the reference standard. We also compared simplicity (from the technicians’ point of view), time taken to obtain results (rank-sum testing), specificity and Kappa statistics of the two methods.

**Results:**

The modified REMA, which is a one-step procedure, was found to be simpler to perform and results were obtained in a significantly shorter time (5 versus 9 days, p < 0.0001) compared to the classic REMA due to addition of indicator and strain at the same time. The specificity of the modified REMA was low {46.8% (35.5% - 58.4%) for RIF and 13.9% (7.2% - 23.5%) for INH}. Kappa statistics were 16.0% for RIF and 2.0% for INH. Low specificity and kappa statistics are due to indicator reduction by the strains before complete drug activity.

**Conclusion:**

Although modified REMA is faster and simpler compared to classic REMA, it is not reliable for MTB drug susceptibility testing.

## Background

The global emergence of drug resistant tuberculosis (DR-TB) underscores the need for rapid detection of drug resistance. Standard agar proportion methods are accurate and low-cost, but take weeks to obtain a result. The GeneXpert MTB/RIF molecular assay [1], which has been endorsed by World Health Organization (WHO) [2], is sensitive, rapid and relatively easy to use. However, it remains costly in resource-limited settings [3,4].

Colorimetric methods of drug susceptibility testing produce results more quickly than standard culture methods [5] and are less costly than molecular methods [2]. For example, the resazurin microtiter assay (REMA), which relies on an oxidation-reduction reaction to induce a blue to pink color change in the presence of live bacteria [6-8], was endorsed by WHO for *Mycobacterium tuberculosis* (MTB) drug sensitivity testing (DST) [2]. Sarker *et al.* have proposed a modification of the classic REMA, to simplify procedures and enable more rapid susceptibility results [9]. The modified REMA shortens the protocol by addition of the isolate and redox indicators at the same time, and has been validated for *Staphylococcus aureus*. However, it has not been evaluated for detection of drug resistance with MTB. We evaluated the performance of the modified REMA for detecting isoniazid (INH) and rifampicin (RIF) susceptibility of MTB strains, using the classic REMA as the reference standard.

### Methods and materials, results, and discussion

We tested archived MTB strains from the National Tuberculosis Reference Laboratory in Kampala, and Epicentre Mbarara Research Centre in Mbarara, Uganda. MTB strains were grown in Middlebrook 7H9 broth (Difco Laboratories, MI, USA) 70 supplemented with 10% albumin– dextrose complex, 0.5% (v/v) glycerol and 0.1% (w/v) casitone and diluted 1:10 of 1.0 McFarland turbidity using Middlebrook 7H9-S broth [10].

**Figure 1:**
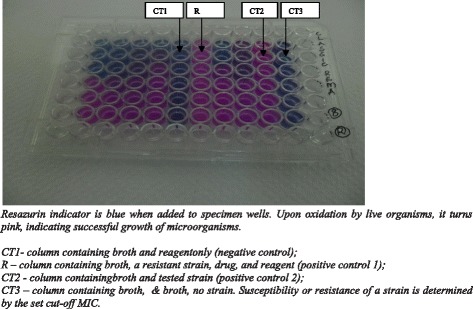
**A REMA plate showing color change.**

We first defined the genetic identity and drug susceptibility for the strains using the MTBDR-Plus Version 2 (HAIN Lifescience, Nehren Germany). We then grew the strains with REMA reagents using both the classic REMA and modified REMA methods. For the classic REMA, we followed standard protocols, as described previously [7,11,12]. Briefly, we incubated the strain-drug mixtures at 37°C for seven days, then added 0.02% resazurin indicator and recorded any color changes (from blue to pink) after 48 hours. In contrast, for the modified REMA [9], we added the strain, drugs and indicator (0.02%) at the same time, incubated at 37°C, and assessed for color changes daily. We measured minimum inhibitory concentrations (MICs) of INH and RIF (Sigma-Aldrich, Milwaukee, WI, USA) [10] by serially diluting the drugs. We defined the MIC for each strain as the lowest concentration of a drug that had no color change (Figure 1). Laboratory technicians recorded data on time taken from culture to DST results.

We categorized strains as sensitive or resistant to RIF and INH by MIC thresholds of ≤0.5 and >0.5; and ≤0.25 and >0.25, respectively [10]. We calculated the sensitivity and specificity of INH and RIF DST by the modified REMA, comparing it to the classic REMA and Hain MTBDR-Plus assays as the reference standards. We also compared paired MIC results of the classic and modified REMA using kappa testing. Finally, we compared time from culture to DST results between the classic and modified REMA tests using non-parametric rank-sum testing. We used Stata version 12.0 (College Station, Texas, USA) for all statistical analyses.

The study was approved by the Institutional Review Committee of Mbarara University of Science and Technology (reference no. 14/09-12), and permission to conduct the study was granted by Uganda National Council for Science and Technology (ref. HS 1312).

All 81 strains tested were confirmed as MTB by the MDR-TB Hain assay. Of these, 78 were susceptible to both RIF and INH, one was mono resistant to RIF, one was mono resistant to INH, and one was resistant to both INH and RIF (MDR). Classic REMA results were in perfect agreement with the MTBDR Plus Hain assay results.

Compared to classic REMA, sensitivity and specificity of the modified REMA were 100% (2/2, 15.8-100) and 13.9% (11/79, 7.16-23.5) for INH, and 100% (2/2, 15.8- 100) and 46.8% (37/79, 35.5-58.4) for RIF (Table 1). Because the classic REMA and Hain assays were in perfect agreement, our estimates were similar with both reference standards. Comparative MIC results for the classic REMA and modified REMA for paired MTB strains were significantly different for both RIF (k = 0.16) (Table 2) and INH (k = 0.02) (Table 3). For the modified REMA assay, 30/81 (37%) and 64/81 (79%) strains grew in the maximum concentration of rifampicin and isoniazid, respectively. Laboratory technologists found the modified REMA subjectively easier to perform compared to the classic REMA, and modified REMA yielded faster results (median time to results was 5 versus 9 days; P < 0.0001).

**Table 1:** **Diagnostic accuracy of the classic and modified resazurin microtiter assay versus the MTBDR Plus Hain assay for detection of drug resistant** ***Mycobacterium tuberculosis***

	**Sensitivity**		**Specificity**	
	**x/y (%)**	**95% CI**	**x/y (%)**	**95% CI**
RIF				
Classic REMA	2/2 (100)	15.8 - 100	79/79 (100)	95.4 - 100
Modified REMA	2/2 (100)	15.8 - 100	37/79 (46.8)	35.5 - 58.4
INH				
Classic REMA	2/2 (100)	15.8 - 100	79/79 (100)	95.4 - 100
Modified REMA	2/2 (100)	15.8 - 100	11/79 (13.9)	7.2 - 23.5

*RIF: rifampicin: INH- isoniazid; REMA:* resazurin microtiter assay.

**Table 2:** **Paired comparison of minimum inhibitor concentrations of Rifampicin for the modified and classic resazurin microtiter assays for detection of drug susceptibility in** ***Mycobacterium tuberculosis***

	**Rifampicin**							
			**Modified REMA MIC**					
	**MIC**	**0.0625**	**0.125**	**0.25**	**0.5**	**1**	**2**	**≥4**
Classic REMA MIC	0.0625	6	4	6	4	4	1	2
	0.125	2	0	0	9	2	1	1
	0.25	1	0	1	3	2	0	8
	0.5	0	0	0	1	2	0	19
	1	0	0	0	0	1	0	0
	2	0	0	0	0	0	1	0
	≥4	0	0	0	0	0	0	0

κ = 0.16.

**Table 3:** **Paired comparison of minimum inhibitor concentrations of Isoniazid for the modified and classic resazurin microtiter assays for detection of drug susceptibility in** ***Mycobacterium tuberculosis***

	**Isoniazid**							
			**Modified REMA MIC**					
	**MIC**	**0.03125**	**0.0625**	**0.125**	**0.25**	**0.5**	**1**	**≥2**
Classic REMA MIC	0.03125	1	2	3	1	1	3	14
	0.0625	0	1	1	0	0	0	12
	0.125	0	0	0	1	0	0	29
	0.25	1	0	0	0	0	0	9
	0.5	0	0	0	0	1	0	0
	1	0	0	0	0	0	1	0
	2	0	0	0	0	0	0	0

κ = 0.02.

*REMA: Resazurin microtiter assay; MIC: minimum inhibitory concentration measured in mg/ml*.

In summary we found that the modified REMA is a rapid and simple DST for MTB. Unfortunately, it has unacceptably poor specificity for detection of both RIF and INH resistance, limiting its applicability. The poor performance of the modified REMA can likely be explained by the requirement to add all reagents simultaneously and the slow growth patterns of MTB. Both INH and RIF primarily target actively dividing cells in the log growth phase. MTB is slow growing, therefore, addition of the indicator simultaneously with the drug might allow the enzymatic reaction of the indicator before the antimycobacterial agents have ample time to inhibit growth. Because the reduction of resazurin is an irreversible reaction [2,6] a susceptible strain may be falsely indicated as resistant. This same phenomenon would not be expected to occur with faster growing organisms with a shorter log phase, for example, *S. aureus*. Thus, while reducing time to results, the early mixing step likely contributes to low specificity. Alternative manipulations of the classic REMA assay should be pursued to help decrease time for DST results while enabling accurate results. In this study, we provide data to suggest that the resazurin indicator does not inhibit MTB growth, as has been previously speculated. Therefore, to exploit the short time required to complete the modified REMA assay, future work should evaluate its utility for rapid diagnosis of TB from sputum samples, without a focus on drug suceptibility.

## Conclusion

Although modified REMA is faster and simpler than the classic REMA, it is not reliable for MTB DST testing due to poor specificity for detection of both INH and RIF resistance. It should not be recommended as a DST for MTB, although its utility as a rapid diagnostic test for MTB in sputum samples should be further evaluated.

## References

[1] Boehme CC, Nabeta P, Hillemann D, Nicol MP, Shenai S, Krapp F, Allen J, Tahirli R, Blakemore R, Rustomjee R, Milovic A, Jones M, O'Brien SM, Persing DH, Ruesch- Gerdes S, Gotuzzo E, Rodrigues C, Alland D, Perkins MD (2010). Rapid molecular detection of tuberculosis and rifampin resistance. N Engl J Med.

[2] Palomino JC, Martin A, Camacho M, Guerra H, Swings J, Portaels F (2002). Resazurin microtiter assay plate: simple and inexpensive method for detection of drug resistance in Mycobacterium tuberculosis. Antimicrob Agents Chemother.

[3] Angeby KA, Klintz L, Hoffner SE (2002). Rapid and inexpensive drug susceptibility testing of Mycobacterium tuberculosis with a nitrate reductase assay. J Clin Microbiol.

[4] Martin A, Montoro E, Lemus D, Simboli N, Morcillo N, Velasco M, Chauca J, Barrera L, Ritacco V, Portaels F, Palomino JC (2005). Multicenter evaluation of the nitrate reductase assay for drug resistance detection of Mycobacterium tuberculosis. J Microbiol Methods.

[5] Martin A, Portaels F, Palomino JC (2007). Colorimetric redox-indicator methods for the rapid detection of multidrug resistance in *Mycobacterium tuberculosis*: a systematic review and meta-analysis. J Antimicrob Chemother.

[6] Pfaller MA, Vu Q, Lancaster M, Espinel-Ingroff A, Fothergill A, Grant C, McGinnis MR, Pasarell L, Rinaldi MG, Steele-Moore L (1994). Multisite reproducibility of colorimetric broth microdilution method for antifungal susceptibility testing of yeast isolates. J Clin Microbiol.

[7] Pital A, Pital RC, Leise JM (1958). A rapid method for determining the drug susceptibility of Mycobacterium tuberculosis. Am Rev Tuberc.

[8] Boum Y, Orikiriza P, Rojas-Ponce G, Riera-Montes M, Atwine D, Nansumba M, Bazira J, Tuyakira E, De Beaudrap P, Bonnet M, Page AL (2013). Use of colorimetric culture methods for detection of Mycobacterium tuberculosis complex isolates from sputum samples in resource-limited settings. J Clin Microbiol.

[9] Sarker SD, Nahar L, Kumarasamy Y (2007). Microtiter plate-based antibacterial assay incorporating resazurin as an indicator of cell growth, and its application in the in vitro antibacterial screening of phytochemicals. Methods.

[10] Martin A, Palomino JC (2012). Procedure manual: Drug susceptibility testing for Mycobacterium tuberculosis. Procedure Manual.

[11] Yajko DM, Madej JJ, Lancaster MV, Sanders CA, Cawthon VL, Gee B, Babst A, Hadley WK (1995). Colorimetric method for determining MICs of antimicrobial agents for Mycobacterium tuberculosis. J Clin Microbiol.

[12] Drummond JA, Waigh DR (2000). Recent research developments. Phytochem.

